# Cloning and Characterization of *TaSAP7-A*, a Member of the Stress-Associated Protein Family in Common Wheat

**DOI:** 10.3389/fpls.2021.609351

**Published:** 2021-03-22

**Authors:** Wenlu Li, Yixue Wang, Runzhi Li, Xiaoping Chang, Xiangyang Yuan, Ruilian Jing

**Affiliations:** ^1^College of Agronomy, Shanxi Agricultural University, Jinzhong, China; ^2^National Key Facility for Crop Gene Resources and Genetic Improvement, Institute of Crop Science, Chinese Academy of Agricultural Sciences, Beijing, China; ^3^College of Life Sciences, Shanxi Agricultural University, Jinzhong, China

**Keywords:** *TaSAP7-A*, abiotic stress, chlorophyll content, *TaS10B*, wheat

## Abstract

Stress association proteins (SAPs) are A20/AN1 zinc-finger domain proteins, which play important roles in plant adaptation to abiotic stress and plant development. The functions of SAPs in some plants were reported, but little is known about it in wheat (*Triticum aestivum* L.). In this study, we characterized a novel 2AN1-type stress association protein gene *TaSAP7-A*, which was mapped to chromosome 5A in wheat. Subcellular localization indicated that TaSAP7-A was distributed in the nucleus and cytoplasm. Unlike previously known A20/AN1-type SAP genes, *TaSAP7-A* was negatively regulated to abiotic stress tolerance. Overexpressing *TaSAP7-A Arabidopsis* lines were hypersensitive to ABA, osmotic and salt stress at germination stage and post-germination stage. Overexpression of *TaSAP7-A Arabidopsis* plants accelerated the detached leaves’ chlorophyll degradation. Association analysis of *TaSAP7-A* haplotypes and agronomic traits showed that *Hap-5A-2* was significantly associated with higher chlorophyll content at jointing stage and grain-filling stage. These results jointly revealed that *TaSAP7-A* is related to the chlorophyll content in the leaves of *Arabidopsis* and wheat. Both *in vivo* and *in vitro* experiments demonstrated that TaSAP7-A interacted with TaS10B, which was the component of regulatory subunit in 26S proteasome. In general, *TaSAP7-A* was a regulator of chlorophyll content, and favorable haplotypes should be helpful for improving plant chlorophyll content and grain yield of wheat.

## Introduction

Plants are continuously challenged by environmental stresses, including extreme temperature, drought, and high salinity. In order to cope with these environmental stresses, plants have evolved a series of reaction mechanisms for survival, which induce expression of various stress-related genes for adaption to the surrounding environment (Kasuga et al., 1999; Hirayama and Shinozaki, 2010). Regulators of stress response, such as dehydration-responsive element-binding proteins (DREBs), C-repeat-binding factors (CBFs), heat shock factor/proteins (HSF/HSPs), and NAC domain-containing transcription factor, are highly conserved and extensively exist in plants. They have been used as favorable targets to improve abiotic stress tolerance in a variety of crop plants (Akhtar et al., 2012; Nakashima et al., 2012; Scharf et al., 2012; Jacob et al., 2017). The zinc-finger proteins (ZFPs) are a group of multiple functions regulators in plants and characterized by zinc binding motif composed of conserved cysteines, histidine, and zinc atoms. They can control the expression of many target genes via binding specific targeting non-coding DNA regions called *cis*-acting elements in the promoters of target genes and then regulate plant growth, development, and stress responses (Jan et al., 2013; Zhang et al., 2014; Baek et al., 2015; Wang et al., 2019).

Stress-associated proteins (SAPs) are a class of zinc finger proteins including A20/AN1 domains. *SAP* genes are widely distributed in many plant species, such as rice, maize, tomato, soybean, cucumber, *Arabidopsis*, *Medicago truncatula*, *Aeluropus littpralis*, and *Sorghum bicolor* (Vij and Tyagi, 2006; Solanke et al., 2009; Gimeno Gilles et al., 2011; Xuan et al., 2011; Wang et al., 2013; Zhang et al., 2019; Lai et al., 2020). *SAP* genes are known to play important roles in stress responses in plants, and majority of them have been reported to be stress inducible. Overexpressed *SAP* gene transgenic plants display changes in abiotic stress tolerance to salinity, drought, cold, heat, and toxic metals (Giri et al., 2013). In plants, the first *SAP* gene *OsiSAP1* was induced after different types of stresses as well as abscisic acid (ABA). Overexpression of *OsiSAP1* enhanced stress tolerance in tobacco at the germination and seedling stage (Mukhopadhyay et al., 2004). *OsiSAP8* also was a multiple stress inducible gene, and transgenic rice plants were tolerant to salt and drought during anthesis stage without any yield penalty compared with unstressed transgenic plants (Kanneganti and Gupta, 2008). *AtSAP5* improved tolerance to drought and heat stress by upregulating the expression of endogenous stress-responsive genes in transgenic cotton (Hozain et al., 2012). *AtSAP10* showed differential regulation by heavy metals. Overexpression of *AtSAP10* in *Arabidopsis* conferred strong tolerance to heavy metals such as nickel, manganese, and zinc. Moreover, transgenic *Arabidopsis* grew healthy and green under these stress conditions (Dixit and Dhankher, 2011). *OsSAP16*-overexpressed mutants had higher intrinsic water use efficiency by regulating the expression of a set of stress-associated genes in rice (Wang et al., 2016). *AtSAP13* overexpression lines showed strong tolerance to toxic metals, drought, and salt stress (Dixit et al., 2018). In addition, *SAP* genes in other plants such as *AlSAP*, *MdSAP15*, *MtSAP1*, *MusaSAP1*, and *PtSAP13*, were reported as positive regulators in different stress responses (Ben Saad et al., 2010, 2012; Charrier et al., 2012, 2013; Sreedharan et al., 2012; Dong et al., 2018; Li J. et al., 2019).

Wheat (*Triticum aestivum* L.) is one of the most important cereal crops in the world. There are abundant allelic variations in wheat germplasm. Association analysis is regarded as an effective way to identify the favorite allelic variations. In this study, we reported a novel 2AN1-type stress association protein gene *TaSAP7-A*, which played a negative role in abiotic stress. The phenotypes of transgenic *Arabidopsis* and association analysis jointly indicated that it related to chlorophyll content. TaS10B, a regulatory subunit of 26S proteasome, was screened and confirmed to interact with TaSAP7-A.

## Materials and Methods

### Plant Material, Growth Condition, and Measurement of Agronomic Trait

Drought-tolerant common wheat (*Trticum aestivum* L.) cultivar Hanxuan 10 was used for cloning gene *TaSAP7-A* and expression analysis in stress condition. A set of nulli-tetrasomic lines of Chinese Spring were used for chromosomal location of *TaSAP7-A*. Thirty-two highly diverse accessions were used to identify nucleotide polymorphism of *TaSAP7-A*. A natural population (323 winter wheat accessions) was used for association analysis of *TaSAP7-A* haplotypes and agronomic traits. A wheat 660K SNP array, consisting of 630,517 SNPs, was used to genotype all 323 accessions. By removing nucleotide variations with missing rates ≥0.2 and minor allele frequency (<0.05), 395,681 SNPs were applied to detect the structure of the natural population by the software STRUCTURE 2.3.4 (Li L. et al., 2019). The natural population was sown at Shunyi (40°23′ N; 116°56′ E) and Changping (40°13′ N; 116°13′ E), Beijing, China, over two growth cycles (2014–2016). Four treatments, i.e., well watered (WW), rain fed (drought stressed, DS), well watered in greenhouse (WW + HS, heat stress) and rain-fed in greenhouse (DS + HS), were supplied at each site. The WW plots were irrigated with 750 m^3^ ha^–1^ (75 mm) at the pre-overwintering, booting, flowering, and grain-filling phases, though the DS plots were rain fed (Zhang B. et al., 2017). The amounts of rainfall in the growing seasons were 161 and 173 mm, respectively. Each plot consisted of four 2-m rows with 40 plants in each row. Row-to-row distance was maintained at 0.3 m. Agronomic traits were measured by random selection of five plants to calculate the mean in each accession, including plant height (PH), 1,000-grain weight (TGW), chlorophyll content of flag leaves at jointing (CC-J), and grain filling (CC-F) stages.

*Arabidopsis thaliana* (Columbia ecotype) was chosen for transgenic analysis. *Nicotiana benthamiana* was used for subcellular localization, LCI assays, and BiFC assays. They were grown in a controlled environment chamber at 23°C, with a 12 h/12 h light/dark photoperiod, a light intensity of 120 mmol m^–2^ s^–1^ and 70% relative humidity. *Arabidopsis* seeds were sown on Murashige and Skoog (MS) medium solidified with 0.8% agar, and then vernalized at 4°C for 48 h before culturing in a controlled growth chamber.

### Gene Cloning and Chromosomal Location

To obtain coding and upstream sequence of *TaSAP7-A*, a pair of subgenome A-specific primer *SAP7A-SF/SR* (forward primer, 5′-GATTGATAGACTTATGGTAAG-3′; reverse primer, 5′-CTAATCAGAACATCTTGGAATTC-3′) was designed according to the reference sequence from URGI (Unité de Recherche Génomique Info) website^1^. Thirty-three nulli-tetrasomic lines of Chinese Spring wheat were used for the chromosomal location of *TaSAP7-A*.

### Sequence Alignment and Phylogenetic Analyses

To gather sequences with high similarity, the amino acid sequence of TaSAP7-A was used as a query using BLAST search. The putative sequences were downloaded, and sequence alignments were implemented by DNAMAN. The neighbor-joining phylogenetic tree was built based on 1,000 bootstrap replicates by MEGA 6.

### Subcellular Localization

The full-length ORF of TaSAP7-A was fused upstream of the GFP, which was controlled by the constitutive CaMV 35S promoter in the pCAMBIA1300 vector. The primers *SAP7A-F/R* (forward primer, 5′-TGCTCTAGAATGGCGCGGC GGGGCACGG-3′; reverse primer, 5′-CGGGGTACCGAACATC TTGGAATTCCGG-3′; *Xba*I and *Kpn*I site underlined) were used for subcloning. The constructs were transferred into wheat mesophyll protoplasts by the PEG-mediated method for subcellular localization (Yoo et al., 2007). After incubation at 25°C for 16 h, florescence signals were detected using a laser scanning confocal microscope (Leica TCSNT, Germany). For observation of subcellular localization in tobacco (*Nicotiana benthamiana*) leaf cells, the constructs were transferred into tobacco leaves through *Agrobacterium tumefaciens* (GV3101)-mediated transformation (Liu et al., 2010), and florescence signals were examined after culturing for 3 days at 25°C in a photoperiod of 16 h/8 h light/dark.

### Quantitative Real-Time PCR

Quantitative real-time PCR (qRT-PCR) was performed for gene expression studies. Two-leaf wheat seedlings were subjected to 16.1% polyethylene glycol-6000 (PEG-6000) solution and 250 mM NaCl solution, cultured in low temperature (4°C) condition or sprayed with 50 μM ABA. Leaf samples were harvested at specific points in time. *TaActin* (forward primer, 5′-CTCCCTCACAACAACAACCGC-3′; reverse primer, 5′-TACCAGGAACTTCCATACCAAC-3′) was used as reference gene. The qRT-PCR primers *SAP7A-QF/QR* (forward primer, 5′-AAGCGAGGGGATCGGAAAC-3′; reverse primer, 5′-CGTACGTGCGGTGCTCGGC-3′) were designed to estimate the target gene expression. The qRT-PCR was performed in triplicate with a Roche LightCycler 96 Real-Time PCR System (Roche, Switzerland) using the SYBR Green PCR Master Mix Kit (TaKaRa, Japan). Thermal cycling conditions were pre-incubated at 95°C for 120 s, followed by 95°C for 20 s, 60°C for 20 s, and 72°C for 20 s for 45 cycles. The relative transcription level was calculated using the 2^–ΔΔ^*^*C*^*^T^ method (Schmittgen and Livak, 2008). Five plants were used per treatment, and three biological replications were included in each treatment.

### Generation of *Arabidopsis* Transgenic Plants

The *Agrobacteriums* train GV3101 containing constructs mentioned in the subcellular localization was used for generation of transgenic *Arabidopsis* plants by floral infiltration (Clough and Bent, 1998). Positive T_3_ generation transgenic *Arabidopsis* plants overexpressing *TaSAP7-A* were screened by hygromycin plates and then identified by qRT-PCR. *AtACTIN2* (forward primer, 5′-AGCACTTGCACCAAGCAGCATG-3′; reverse primer, 5′-ACGATTCCTGGACCTGCCTCATC-3′) was used as reference gene. Two T_3_ homozygous transgenic *Arabidopsis* lines with relatively higher expression levels of *TaSAP7-A* were used for phenotypic assays. The expression was performed with three independent biological replicates. Transgenic plants with empty vector and the WT were used as controls.

### Trait Evaluation of Transgenic *Arabidopsis*

To investigate the stress tolerance of transgenic *Arabidopsis* lines, surface-sterilized *Arabidopsis* seeds (49 seeds) were placed on MS medium containing 0.5 μM ABA, 100 mM NaCl, and 200 mM mannitol, respectively. After vernalization for 48 h at 4°C, the seeds were cultured in a controlled growth chamber for 7 days. Photographs were taken, and the proportion of seedlings with cotyledon was calculated. To probe the potential effects of *TaSAP7-A* to leaves, transgenic *Arabidopsis* plants were cultured in soil for 4 weeks. The sixth and seventh rosette leaves were detached and then immersed in ddH_2_O under darkness for 3 days. Chlorophyll contents of detached leaves were measured by methods described in previous papers (Mao et al., 2010). There were three biological replications in the experiments.

### Marker Development and Association Analysis

Markers were developed by two rounds of PCR. The first was to amplify *TaSAP7-A* fragments with the subgenome, a specific primer *SAP7A-SF/SR*. The second was implemented using the first-round PCR products as template with the corresponding primer pairs (*MF1*/*MR1* and *MF2*/*MR2*) and restriction enzymes. On the basis of SNP site (A/G) at −464 bp, a CAPS marker was developed. The primers *MF1*/*MR1* (forward primer, 5′-TCGCTGTGAAGTCAACGTAG-3′; reverse primer, 5′-GTGCAAAGGAAGGCTTGCG-3′) were designed to obtain the PCR product containing the variation site, then digested by *Eco*R I and separated on 2% agarose gel. Another dCAPS marker was developed according to the SNP site (C/T) at −2,044 bp. The primers *MF2*/*MR2* (forward primer, 5′-ATGTATACAAGTTTTGTCTGAATTTAAA-3′; reverse primer, 5′-CCTCCGATCCTTTTTACTTCG-3′, base mismatching underlined) were designed to obtain the PCR product containing the variation site. *MF2* was designed by one base mismatching (G→T), then a restriction enzyme *Swa* I recognition site was produced. The PCR product was digested by *Swa* I and separated on 4% agarose gel. The general linear model (GLM) in TASSEL 2.1 software was used for association analysis. Associations were considered significant at *P* < 0.05. One-way analysis of variance (ANOVA) was conducted by using SPSS 16.0 V.

### Transcriptional Activity and Yeast Two-Hybrid Assay

The yeast (*Saccharomyces cerevisiae*) strain AH109 and GAL4-based Matchmaker Two-Hybrid System (Clontech) were used in transcriptional activity and yeast two-hybrid assays. The full-length ORF of TaSAP7-A and two truncations were cloned into pGBKT7 to produce in-frame fusions to GAL4-binding domain. The two truncations were at positions 1–100 and 101–182 amino acids of TASAP7-A, containing the first and the second AN1 domain, respectively. These constructs were then transformed into yeast strain AH109 and cultured until optical density at 600 nm = 1.0. The yeast suspension was inoculated onto SD/-Trp, SD/-Trp/-His. The pGBKT7 vector was used as the negative control.

A cDNA yeast library constructed from wheat variety Hanxuan 10 was performed on screening-interacting protein of TaSAP7-A. The full-length ORFs of TaSAP7-A and TaS10B were separately amplified with primer pairs *SAP7A-BDF/BDR* (forward primer, 5′-GGAATTCCATATG ATGGCGCGGCGGGGCACG-3′; reverse primer, 5′-CGCGGA TCCTCAGAACATCTTGGAATTCCG-3′; *Nde*I and *Bam*HI site underlined) and *S10B-ADF/ADR* (forward primer, 5′-GGAATTCCATATGATGGCCGAGGCCGACGACG-3′; reverse primer, 5′-CCGGAATTCTTAGTCCTTGCCAAAGTCGG-3′; *Nde*I and *Bam*HI site underlined). The products were cloned into the pGBKT7 and pGADT7 vector, respectively, and then constructs were co-transformed into yeast strain AH109. Transformations were selected on SD/-Trp/-Leu medium. To evaluate protein interactions, the transformations were inoculated onto SD/-Ade/-His/-Trp/-Leu/X-α-gal medium.

### Firefly Luciferase Complementation Imaging Assay

The full-length ORFs of TaSAP7-A and TaS10B were amplified with primer pairs *SAP7A-nLF/nLR* (forward primer, 5′-CGGG GTACCATGGCGCGGCGGGGCACGG-3′; reverse primer, 5′-ACGCGTCGACGAACATCTTGGAATTCCGGAC-3′; *Kpn*I and *Sal*I site underlined) and *S10B-cLF/cLR* (forward primer, 5′-CGGGGTACCATGGCCGAGGCCGACGACG-3′; reverse primer, 5′-ACGCGTCGACGTCCTTGCCAAAGTCGGCGC-3′; *Kpn*I and *Sal*I site underlined) and ligated into the p1300-nLuc and p1300-cLuc vectors, respectively. The constructs were transformed into *Agrobacterium tumefaciens* strain GV3101, and further co-transferred into tobacco leaves. After 2 days, the leaves were sprayed with 1 mM luciferin and kept in darkness for 10 min. A camera fitted with a low-light cooled charge-coupled device was used to capture the luciferase image.

### Bimolecular Fluorescence Complementation Assay

The full-length ORFs of TaSAP7-A and TaS10B were separately amplified with primer pairs *SAP7A-nYF/nYR* (forward primer, 5′-ATGGCGCGGCGGGGCACGG-3′; reverse primer, 5′-GAACATCTTGGAATTCCGGAC-3′) and *S10B-cYF/cYR* (forward primer, 5′-ATGGCCGAGGCCGACGACG-3′; reverse primer, 5′-GTCCTTGCCAAAGTCGGCGC-3′) and then ligated into the entry vector. After that, target genes were transited to BiFC vectors pEarleygate201-nYFP and pEarleygate202-cYFP through Gateway and co-expressed in tobacco epidermal cells through *Agrobacterium tumefaciens* (GV3101)-mediated transient expression. The florescence signal of yellow fluorescent protein (YFP) was detected at 3 days after infiltration.

## Results

### Cloning and Chromosomal Location of *TaSAP7-A*

**FIGURE 1:**
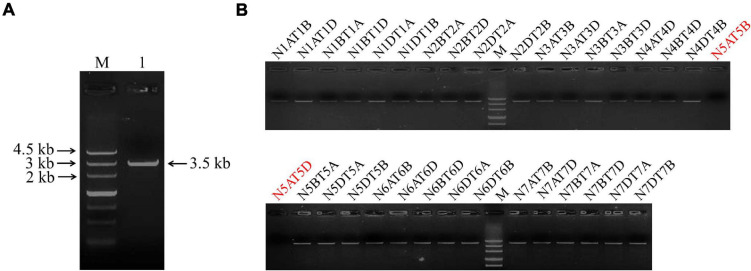
Clone and chromosomal location of *TaSAP7-A*. **(A)** *TaSAP7-A* isolated from Hanxuan 10. Lane 1 is the PCR product of *TaSAP7-A*. **(B)** *TaSAP7-A* was located on chromosome 5A using nulli-tetrasomic lines of Chinese Spring. M is Marker III.

The full length of the *TaSAP7-A* gene (∼3.5 kb) including the coding region (549 bp) and upstream sequence (2,990 bp) was cloned with specific primers *Sap7A-SF* and *Sap7A-SR* from wheat cultivar Hanxuan 10 (Figure 1A). Using a set of nulli-tetrasomic lines of Chinese Spring, *TaSAP7-A* was located on chromosome 5A (Figure 1B), so it has −*A* in name.

### Sequence Analysis of *TaSAP7-A*

**FIGURE 2:**
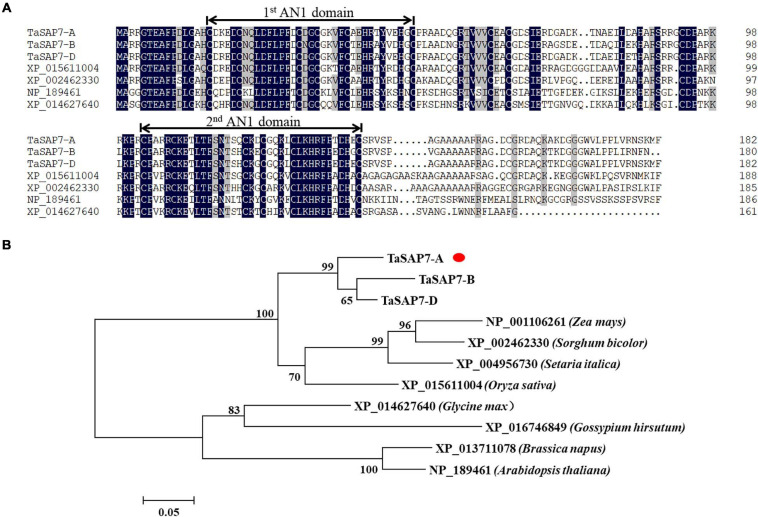
Wheat TaSAP7-A belongs to the stress association proteins (SAPs) protein family. **(A)** Alignment of SAPs from different plant species; Ta from *Triticum aestivum*, XP_015611004 from *Oryza sativa*, XP_002462330 from *Sorghum bicolor*, NP_189461 from *Arabidopsis thaliana*, and XP_014627640 from *Glycine max*. Numbers on the right indicate amino acid position. Amino acid residues with 100% similarity are shown in black background; amino acid residues with no less than 75% similarity are shown in gray background. The conserved AN1 domains are marked above the alignment with lines. **(B)** Phylogenetic tree of SAP proteins. The neighbor-joining tree was built with 1,000 bootstrap replicates. TaSAP7-A are marked with red dots.

*TaSAP7-A* encodes a protein containing 182 amino acids. The amino acid sequence similarities of TaSAP7-A, TaSAP7-B, and TaSAP7-D were 95.03%. In contrast to previously reported SAP family members, which include one A20 and one AN1 domain, TaSAP7-A contains two AN1 domains, both of which are CX(4)CX(9,12)CX(1,2)CX(4)CX(2)HX(5)HXC. There was no intron in the coding region. Two AN1 domains were highly conserved in rice, sorghum, soybean, and *Arabidopsis* (Figure 2A). A neighbor-joining phylogenetic tree was constructed to determine the relationship between TaSAP7-A and their counterparts in other plant species. The TaSAP7-A was classified in the same clade from monocotyledons, including rice, maize, millet, and sorghum (Figure 2B).

### Subcellular Localization of TaSAP7-A

**FIGURE 3:**
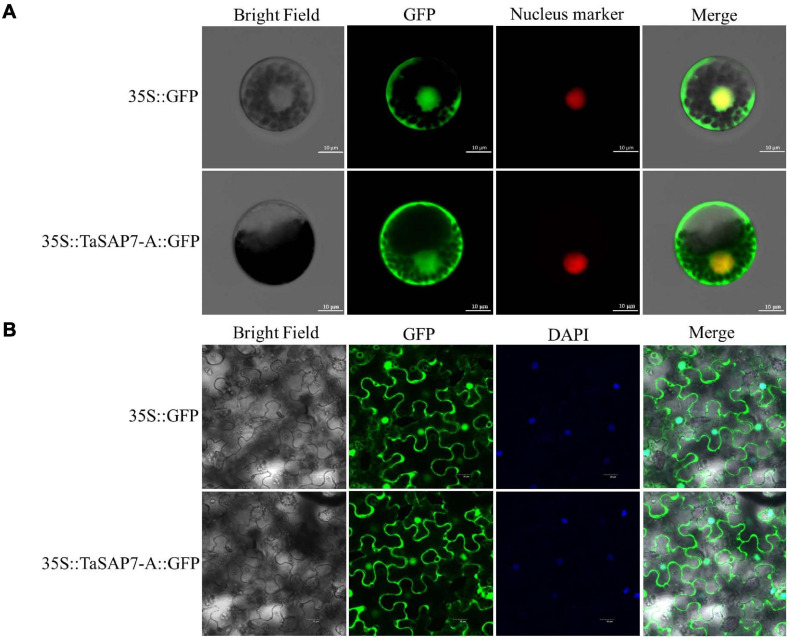
Subcellular localization of TaSAP7-A in wheat protoplasts **(A)** and tobacco leaf cells **(B).** The vector control (35S::GFP) and fusion protein construct 35S::TaSAP7-A::GFP were introduced into wheat protoplast and tobacco leaf cells, respectively. For wheat protoplast transformation, the nucleus marker D53-mCherry was co-transformed into the protoplasts and GFP was detected at 16 h with a laser scanning confocal microscope. For tobacco, GFP was detected at 3 days. Then, 4,6-diamino-2-phenyl indole (DAPI) was used to stain cell nuclei. Scale bars: 10 μm for wheat protoplasts, 20 μm for tobacco leaf cells.

To examine the subcellular localization of TaSAP7-A, full-length *TaSAP7-A* cDNA was fused in-frame to the N-terminus of the GFP coding sequence. TaSAP7-A-GFP fusion protein driven by a CaMV35S promoter was transiently expressed in wheat protoplasts (Figure 3A) and tobacco leaf cells (Figure 3B). Fluorescence was found in both nucleus and cytoplasm.

### Expression Patterns of *TaSAP7-A*

**FIGURE 4:**
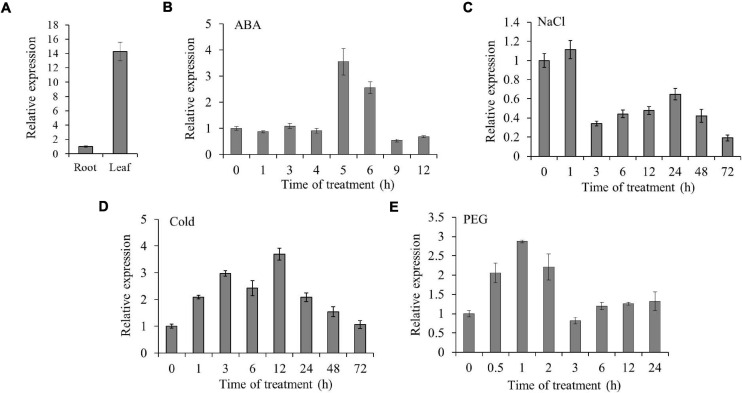
Expression patterns of *TaSAP7-A*. **(A)** Tissue expression of *TaSAP7-A* in wheat seedlings detected by qRT-PCR. **(B–E)** Expression patterns of *TaSAP7-A* in wheat seedlings exposed to 50 μM abscisic acid (ABA), under salt stress (250 mM NaCl), cold stress (4°C) and osmotic stress (16.1% PEG-6000), respectively. The histogram represents mean ± SE of three biological replicates.

To detect the expression of *TaSAP7-A* in different tissues, real-time PCR was performed to analyze the tissue expression at seedling stage in wheat. The expression level of *TaSAP7-A* was 14-fold higher in leaf than root (Figure 4A). Furthermore, the expression patterns of *TaSAP7-A* under multiple stresses were analyzed, including ABA, NaCl, 4°C, and PEG-6000. The *TaSAP7-A* transcripts levels were increased from 5 to 6 h, being 3.5- to 2.5-fold higher after ABA stress (Figure 4B). The expression was slightly increased at 1 h and then decreased by salt stress (∼0.5-fold; Figure 4C). For 4°C treatment, the alteration of expression was gradually increased and then reduced, and maximal levels of transcripts were detected at 12 h (∼3.7-fold; Figure 4D). It was found that expression of *TaSAP7-A* was increased within 2 h after osmotic stress, and levels of upregulation were 2- to 2.9-fold (Figure 4E).

### *TaSAP7-A* Transgenic *Arabidopsis* Plants Are Hypersensitive to ABA and Abiotic Stress

**FIGURE 5:**
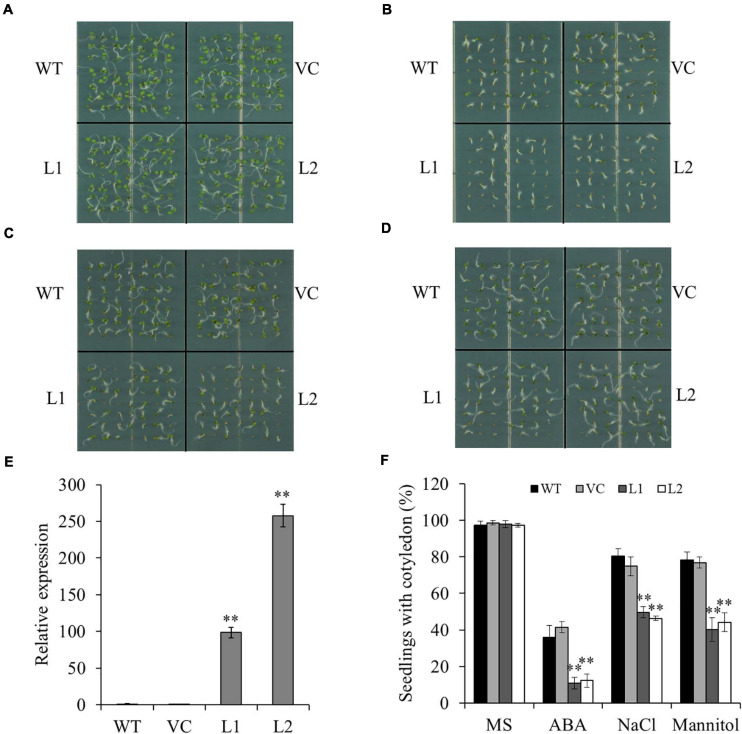
Overexpression of *TaSAP7-A* in *Arabidopsis* enhances sensitivity to ABA, high salinity and osmotic stress. Phenotypes of *TaSAP7-A* overexpressing *Arabidopsis* on MS solid medium **(A)**, MS containing 0.5 μM ABA **(B)**, MS containing 100 mM NaCl **(C)** and MS containing 200 mM mannitol **(D)**. **(E)** The expression level of *TaSAP7-A* in two transgenic lines (L1 and L2). **(F)** Comparison of seedlings with cotyledon of transgenic lines treated with 0.5 μM ABA, 100 mM NaCl and 200 mM mannitol. WT, wild type; VC, plants transformed with the empty pCAMBIA1300 vector; L1 and L2, transgenic lines. The histogram represents mean ± SE of three biological replicates. ^∗∗^*P* < 0.01.

To study the biological function of *TaSAP7-A*, transgenic *Arabidopsis* plants with overexpression of *TaSAP7-A* were generated. Two independent transgenic lines (L1 and L2) with different expression levels of *TaSAP7-A* were selected for further studies (Figure 5E). The wild type (WT) and vector control (VC) were used as controls. Under normal conditions, there was no significant difference between transgenic lines (97.3–98.0%) and the controls (97.3–98.6%; Figure 5A). To investigate whether *TaSAP7-A* was involved in the ABA signaling pathway, the sensitivity of transgenic lines to ABA was tested during seed germination. On the medium containing 0.5 μM ABA, the seedlings with cotyledon of transgenic lines (10.9–12.2%) were significantly reduced when compared with WT and VC (36.1–41.5%; Figure 5B). It was investigated that the ABA hypersensitivity phenotype was correlated with altered expression of ABA-responsive genes, including *AtEm1*, *AtEm6*, *AtRAB18*, and *AtABI5* (Supplementary Figure S1). In addition, we tested the sensitivity of transgenic lines to salt stress and osmotic stress in the germination stage. Under treatment of 100 mM NaCl and 200 mM mannitol, all of WT, VC, and transgenic lines were inhibited by salt stress and osmotic stress, but transgenic lines were more severe than WT and VC (Figures 5C,D). The transgenic lines (40.1–49.7%) showed a significantly lower proportion of seedlings with cotyledon than that of WT and VC (74.8–80.3%; Figure 5F). The seeds of transgenic lines WT and VC were germinated on MS plates for stratification at 4°C for 2 days, and the plates were incubated in a vertical position to allow root growth for 15 days. Compared with WT and VC, the transgenic lines grew weakly and exhibited shorter roots under these stress conditions (Supplementary Figure S2). These results suggested that transgenic lines were more sensitive to ABA, high salinity, and osmotic stress.

### *TaSAP7-A* Transgenic *Arabidopsis* Plants Accelerate Detached Leaves’ Chlorophyll Degradation

**FIGURE 6:**
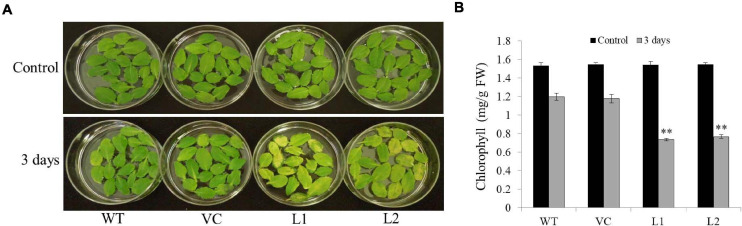
Overexpression *TaSAP7-A* accelerated chlorophyll degradation in detached leaves of *Arabidopsis*. **(A)** Phenotypes of overexpressing *TaSAP7-A Arabidopsis* detached leaves under darkness for 3 days. **(B)** Comparison of chlorophyll content in detached leaves of transgenic, WT and VC *Arabidopsis* plants under darkness for 3 days. The histogram represents mean ± SE of three biological replicates. ^∗∗^*P* < 0.01.

Under darkness, detached leaves turned yellow as time passed; however, the transgenic line leaves changed faster than those of the WT and VC (Figure 6A). Chlorophyll contents in transgenic line leaves (0.73–0.77 mg/g FW) were significantly lower than those of WT and VC (1.18–1.2 mg/g FW) under darkness for 3 days (Figure 6B). These results indicated that *TaSAP7-A* might involve in chlorophyll degradation of detached leaves.

### Polymorphisms and Functional Marker Development of *TaSAP7-A*

**FIGURE 7:**
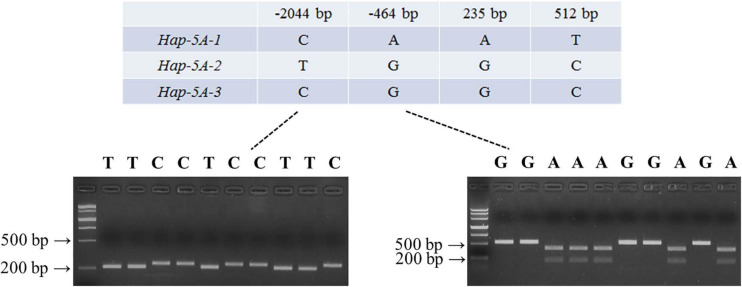
Marker development of *TaSAP7-A*. Partial single nucleotide polymorphisms in three *TaSAP7-A* haplotypes identified among 32 wheat accessions. PCR products of dCAPS (left) and CAPS (right) markers were restrictively digested by *Swal* I and *Eco*R I, respectively. M is Marker III.

Nucleotide polymorphisms in *TaSAP7-A* coding and upstream regions were detected by using 32 diverse accessions (Supplementary Table S1). There were two SNPs (single-nucleotide polymorphisms) in the coding region, 33 SNPs, and four InDel (insertion–deletion) in upstream sequence (Supplementary Table S2), respectively. Two SNPs at 235 bp (A/G) and 512 bp (T/C) in the coding region led to changes in two amino acid residues (Asn79–Asp79, Val171–Ala171). In the two SNPs in the coding region exists the linkage to the SNP at –464 bp in the upstream sequence. On the basis of SNPs (–464 and –2,044 bp) of *TaSAP7-A*, a CAPS marker and dCAPS markers were developed, separately. In the CAPS marker, the PCR product of 529 bp containing the variation site was amplified by the primers *MF1*/*MR1*. The A genotype accessions digested by *Eco*R I exhibited two bands (373 and 156 bp), while the G genotype accessions showed only one band (529 bp) when the digested PCR products on 2% agarose gel were separated. Similarly, the PCR product of 236 bp containing the variation site was amplified by the primers *MF2*/*MR2* in the dCAPS marker. The T genotype accessions digested by *Swa* I exhibited 211 and 25 bp bands, while the C genotype accessions showed a 236 bp band when the digested PCR products on 4% agarose gel were separated. Three haplotype named *Hap-5A-1*, *Hap-5A-2*, and *Hap-5A-3* were identified in the natural population by the markers (Figure 7).

### Association Analysis of *TaSAP7-A* Haplotypes and Agronomic Traits

**FIGURE 8:**
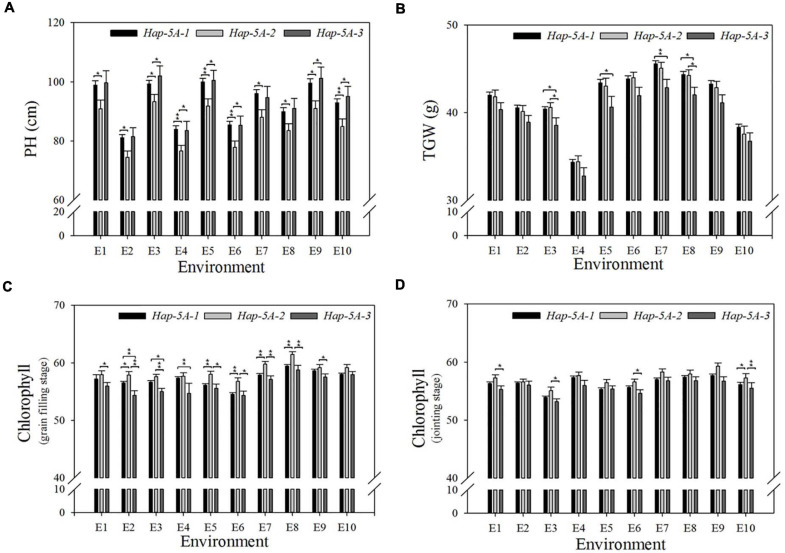
Phenotypic comparisons of three *TaSAP7-A* haplotypes in 10 environments. **(A)** Plant height. **(B)** 1,000 grain weight. **(C)** Chlorophyll content at jointing stage. **(D)** Chlorophyll content at grain filling stage. *P < 0.05, **P < 0.01. E1, 2014-SY-WW; E2, 2014-SY-DS; E3, 2014-SY-WW+HS; E4, 2014-SY-DS+HS; E5, 2015-CP-WW; E6, 2015-CP-DS; E7, 2015-SY-WW; E8, 2015-SY-DS; E9, 2015-SY-WW+HS; E10, 2015-SY-DS+HS. CP, Changping; SY, Shunyi; WW, well-watered; DS, drought-stressed; HS, heat-stressed. Error bars denote 1 SE.

A natural population consisting of 323 accessions was used for association analysis (Supplementary Table S3). The proportion of three haplotypes was 74.3, 15.2, and 10.5%, respectively. Significant association was noted between *TaSAP7-A* haplotypes and agronomic traits, including plant height (PH), 1,000-grain weight (TGW), and chlorophyll content at the jointing stage and grain filling stage in 8, 8, 5, and 8 out of 10 environments (year × site × water regimes × heat; Table 1), respectively. Compared with the other two haplotypes, *Hap-5A-2* was a superior haplotype for shorter PH, but with higher chlorophyll content at the jointing stage and grain filling stage, and higher TGW. The plant height of *Hap-5A-2* was 5.9–10.3 cm lower than those of *Hap-5A-1* and *Hap-5A-3*. The chlorophyll content of *Hap-5A-2* at the jointing stage and grain filling stage was 0.5–2.6 and 1.2–3.5 (SPAD value) higher than that of *Hap-5A-3*, respectively. The TGW of *Hap-5A-2* was 0.8–2.4 g higher than that of *Hap-5A-3* (Figure 8).

**TABLE 1:** *TaSAP7-A* haplotypes associations with agronomic traits in 10 environments.

**Year**	**Site**	**Environment**	**PH *p-value***	**TGW *p-value***	**CC-J *p-value***	**CC-GF *p-value***
2014	SY	WW	0.0193*	n.s.	0.032*	n.s.
	SY	DS	0.0306*	0.0297*	n.s.	1.77E-04***
	SY	WW + HS	0.0069**	0.0391*	0.0264*	8.58E-04***
	SY	DS + HS	n.s.	0.0426*	0.0074**	5.04E-04***
2015	CP	WW	0.0173*	0.0036**	n.s.	0.0314*
	CP	DS	0.0341*	0.0365*	0.0325*	0.0179*
	SY	WW	n.s.	0.0083**	n.s.	0.0046***
	SY	DS	0.0356*	0.0125*	n.s.	0.0038***
	SY	WW + HS	0.0054**	n.s.	0.0047***	0.0372*
	SY	DS + HS	0.0027***	0.0201*	n.s.	n.s.

PH, plant height; TGW, 1,000 grain weight; CC-J, Chlorophyll content at jointing stage; CC-GF, Chlorophyll content at grain filling stage; n.s., not significant; *P < 0.05, **P < 0.01, and ***P < 0.005, respectively. The 10 environments were at Changping (CP) and Shunyi (SY) under well-watered (WW), drought-stressed (DS) and heat-stressed (HS) conditions in 2014 and 2015.

### Transcriptional Activation Activity and Interacting Proteins of TaSAP7-A

**FIGURE 9:**
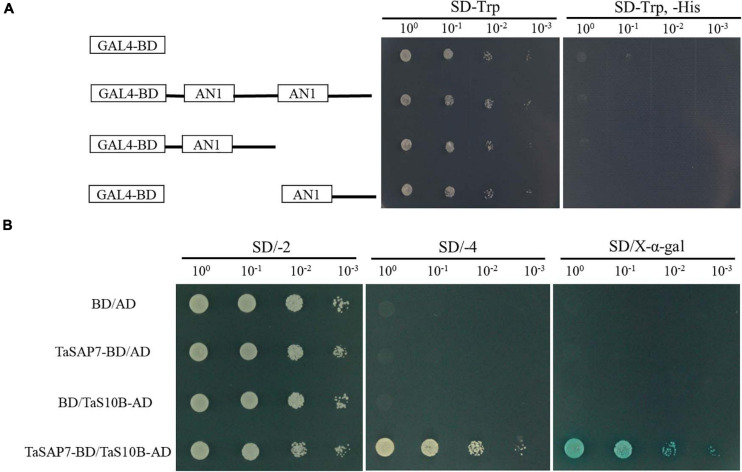
Transcriptional activation activity assay and TaSAP7-A interacts with TaS10B in yeast system. **(A)** According to the amino acid position of the conserved domain, transcriptional activation activity of TaSAP7-A of the full-length and two truncations. **(B)** The transformants were placed on SD/−2 (−Trp, −Leu) medium to examine growth. Protein-protein interactions were assessed on SD/−4 (−Ade, −His, −Trp, −Leu) medium and further confirmed by monitoring α-galactosidase activity.

The structure of TaSAP7-A was a zinc finger protein composed of two AN1 domains. For transcriptional activation activity assays, the AH109 yeast cells containing TaSAP7-A of full-length fusion protein and two-truncation fusion protein grew well on SD/-Trp, but not on SD/-Trp/-His. The result indicated that both TaSAP7-A of full-length and two truncations had no transcriptional activation activity (Figure 9A).

The full-length of TaSAP7-A was used as the bait to screen a yeast two-hybrid cDNA library of wheat to identify TaSAP7-A-interacting proteins. Yeast two-hybrid screening yielded six candidate clones. One of them, a 26S protease regulatory subunit S10B protein designated TaS10B was found to interact with TaSAP7-A using yeast two-hybrid analysis (Figure 9B). TaS10B possesses an AAA domain and codes 400 amino acids with a predicted p*I* of 7.03 and a molecular weight of 44.76 kDa.

### LCI Assays and BiFC Assays

**FIGURE 10:**
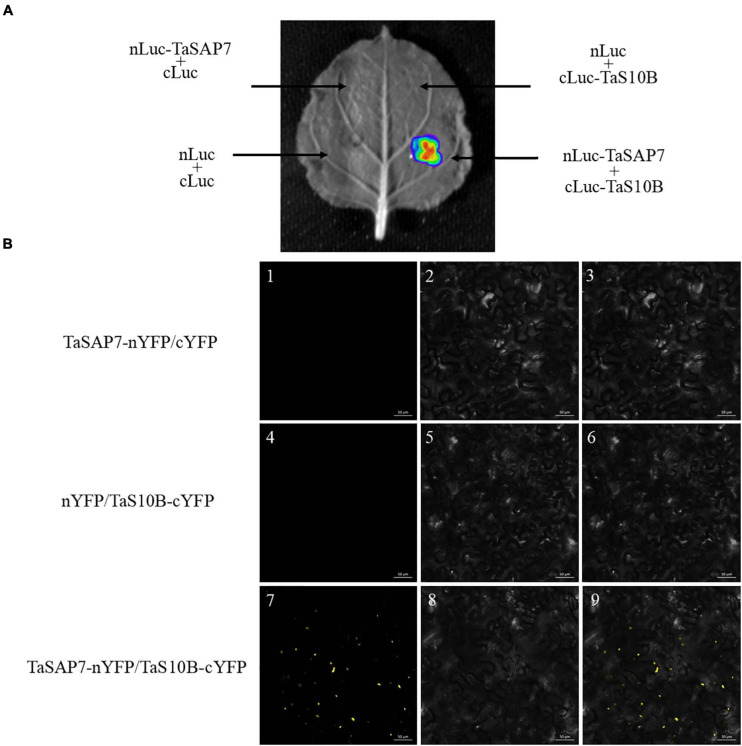
TaSAP7-A interacts with TaS10B in plant system. **(A)** LCI assays showed that TaSAP7-A interacted with TaS10B in tobacco leaves which were infiltrated with *Agrobacterium tumefaciens* strain GV3101 containing the indicated constructs. The signals were collected at 48 h after infiltration. **(B)** BiFC assays made clear that interaction location of TaSAP7-A and TaS10B exist in the cytoplasm of tobacco. Strain GV3101 carrying the indicated constructs were infiltrated into tobacco leaves. Fluorescence was detected 3 days after transformation. Images are in dark field (1, 4, 7), bright field (2, 5, 8), and combined (3, 6, 9). Scale bar, 50 μm.

To define the interaction of TaSAP7-A and TaS10B in a plant system, firefly luciferase (LUC) complementation imaging (LCI) assays were conducted. TaSAP7-A was fused to the N-terminal part of LUC to produce the TaSAP7-A-nLUC construct, and TaS10B was fused to the C-terminal part of LUC to generate the cLUC-TaS10B construct. When TaSAP7-A, -nLUC, and cLUC-TaS10B were co-infiltrated into the tobacco leaf, strong LUC activity was observed (Figure 10A).

Furthermore, BiFC (bimolecular fluorescence complementation) assay was performed to test the location of the interaction. Yellow florescence spots were detected only when TaSAP7-A-nYFP and TaS10B-cYFP were co-expressed in tobacco leaf cells (Figure 10B). It was speculated that these spots could be a protease complex interaction with TaSAP7-A in the cytoplasm.

## Discussion

The growth and development of crops are often impacted by abiotic stress, which leads to yield reduction. Improving stress tolerance without reducing yield has been a great challenge in crop breeding. SAPs are a kind of proteins that contain A20/AN1 zinc-finger domain proteins, which participate in controlling or regulating biological processes. *TaSAP1* and *TaSAP2* were induced by multiple abiotic stresses, and overexpression in *Arabidopsis* enhanced tolerance to drought (Wang, 2011). *TaSAP5* was involved in response to drought stress, and its overexpression in *Arabidopsis* and wheat seedlings increased their tolerance to drought (Zhang N. et al., 2017). *TaSAP17-D*, isolated from subgenome D of wheat, improved salt stress tolerance in transgenic *Arabidopsis* by upregulating the expression of marker genes related to salt stress response (Xu et al., 2018). However, in this study, *TaSAP7-A*, isolated from the subgenome A of wheat, was a negative regulator in the process of abiotic stress tolerance during germination and post-germinative development compared with the majority of *SAP* genes. Recent research reported that the minority of *SAP* genes, such as *ZFP185*, *OsiSAP7*, and *AtSAP9*, acted as a negative regulator of stress signal (Sharma et al., 2015; Zhang et al., 2016; Kang et al., 2017). Interestingly, the 7 d-seedlings of overexpressing *TaSAP7-A* were transplanted to MS medium with mannitol or NaCl for 7 days. There was no significant difference between the transgenic lines and the controls (Supplementary Figure S4). It is speculated that *TaSAP7-A* functions at certain stages of plant development. Plant hormone abscisic acid (ABA) plays a crucial role in plant response to abiotic stress. The environmental stresses induce accumulation of ABA and lead to stress-responsive gene expression via the conserved ABRE and MYC/MYB cis-acting elements (Shinozaki et al., 2003). Our research showed that the overexpression of *TaSAP7-A* transgenic *Arabidopsis* were more sensitive to ABA at the germination stage, and representative ABA-responsive genes were regulated by *TaSAP7-A*. Hence, it was speculated that *TaSAP7-A* joined in ABA-mediated signaling pathway.

Enormous amounts of allelic variations exist in wheat germplasm. Mining favorable alleles and developing their markers are the basis for marker-assisted selection breeding. Association analysis is considered as a powerful approach for identifying functional alleles (Zhang et al., 2013). The two SNPs in the coding region of *TaSAP7-A* led to two amino acid residue change, and they could affect the function of the gene. Previous studies indicated that *TaSAP1-A1* haplotypes significantly associated with agronomic traits, such as 1,000-grain weight, spike length, and number of spikelet per spike. The favored haplotype *TaSAP1-A1 Hap*III was accumulated in wheat-breeding programs (Chang et al., 2013, 2014). The functional marker of *TaSAP7-B* was identified by the dCAPS marker, which was significantly associated with 1,000-grain weight and plant height. The superior allelic variation of *TaSAP7-B* was apparently selected in wheat-breeding programs (Wang et al., 2018). The present developed functional markers of *TaSAP7-A*, which associated with 1,000-grain weight, plant height, and chlorophyll content at the jointing stage and grain-filling stage, could be used in target trait selection in wheat-breeding programs. Wheat genotypes possessing *TaSAP7-A* haplotype *Hap-5A-2* had higher 1,000-grain weight and chlorophyll content, and shorter plant height than those of *Hap-5A-1* and *Hap-5A-3*. The functional markers of *TaSAP7-A* and *TaSAP7-B* both related to 1,000-grain weight and plant height, which implied that the two SAP proteins might have some common functions in wheat.

Despite some *SAP* genes were isolated and identified in various plants, their action mechanism remained largely unknown. SAPs could bind to cis-acting elements to activate the downstream stress-responsive genes. Moreover, they could interact with other proteins such as receptor-like cytoplasmic kinase (RLCK). It was found that A20 domain mediated the interaction of OsSAP1/11 and OsRLCK253, and then OsSAP1/11 was phosphorylated to function (Giri et al., 2011). OsSAP1 interacted with aminotransferase (OsAMTR1) and pathogenesis-related 1a protein (OsSCP) and enhanced tolerance to salt and osmotic stress (Kothari et al., 2016). The ubiquitin/26S proteasome pathway was important in the posttranslational modification in plants. The recent research indicated that SAPs acted as ubiquitin ligase to degrade proteins via ubiquitination. Both AtSAP5 and OsiSAP7 exhibited ubiquitin ligase activity; however, they were positive and negative regulators in the stress response, respectively (Kang et al., 2011; Sharma et al., 2015). TaSAP5 acted as an ubiquitin ligase to mediate DRIP degradation and thus increased DREB2A protein accumulation, which activated the expression of downstream stress-responsive genes (Zhang N. et al., 2017). AtSAP9 interacted with Rad23d, a shuttle factor for the transport of ubiquitinated substrates to the proteasome (Kang et al., 2017). In this study, TaSAP7-A interacted with TaS10B, which was the component of regulatory subunit in 26S proteasome. The expression patterns of *TaS10B* are not exactly similar to that of *TaSAP7-A*; however, *TaS10B* is responsive to abiotic stress conditions (Supplementary Figure S3). TaSAP7-A might promote the protein degradation efficiency by interacting with TaS10B. All in all, as a member of the *SAP* gene family, *TaSAP7-A* was proven as a negative regulator to abiotic stress in wheat first, which enriched a new understanding of the *SAP* genes. It was speculated that *TaSAP7-A* had its unique regulatory pathway in regulating abiotic stress tolerance.

## Data Availability Statement

The datasets generated for this study can be found in the NCBI GenBank accession number for sequencing data of TaSAP7-A: BankIt2400991 Seq1—MW267645.

## Author Contributions

RJ, WL, and YW conceived the idea. WL, YW, XY, and XC performed the experiments. WL, YW, and RL analyzed the data. WL and YW wrote the manuscript. XY and RJ revised the manuscript. All authors contributed to the article and approved the submitted version.

## Conflict of Interest

The authors declare that the research was conducted in the absence of any commercial or financial relationships that could be construed as a potential conflict of interest.

## Footnotes

1 https://urgi.versailles.inra.fr/blast/
