# Supplementary material for: Cloning and Characterization of TaSAP7-A, a Member of the Stress-Associated Protein Family in Common Wheat
Source: Front Plant Sci. 2021 Mar 22;12:609351. doi: 10.3389/fpls.2021.609351 (PMC8020846; doi:10.3389/fpls.2021.609351)
Supplement: Supplementary file 1 [file Data_Sheet_1.doc]

Figure S1.


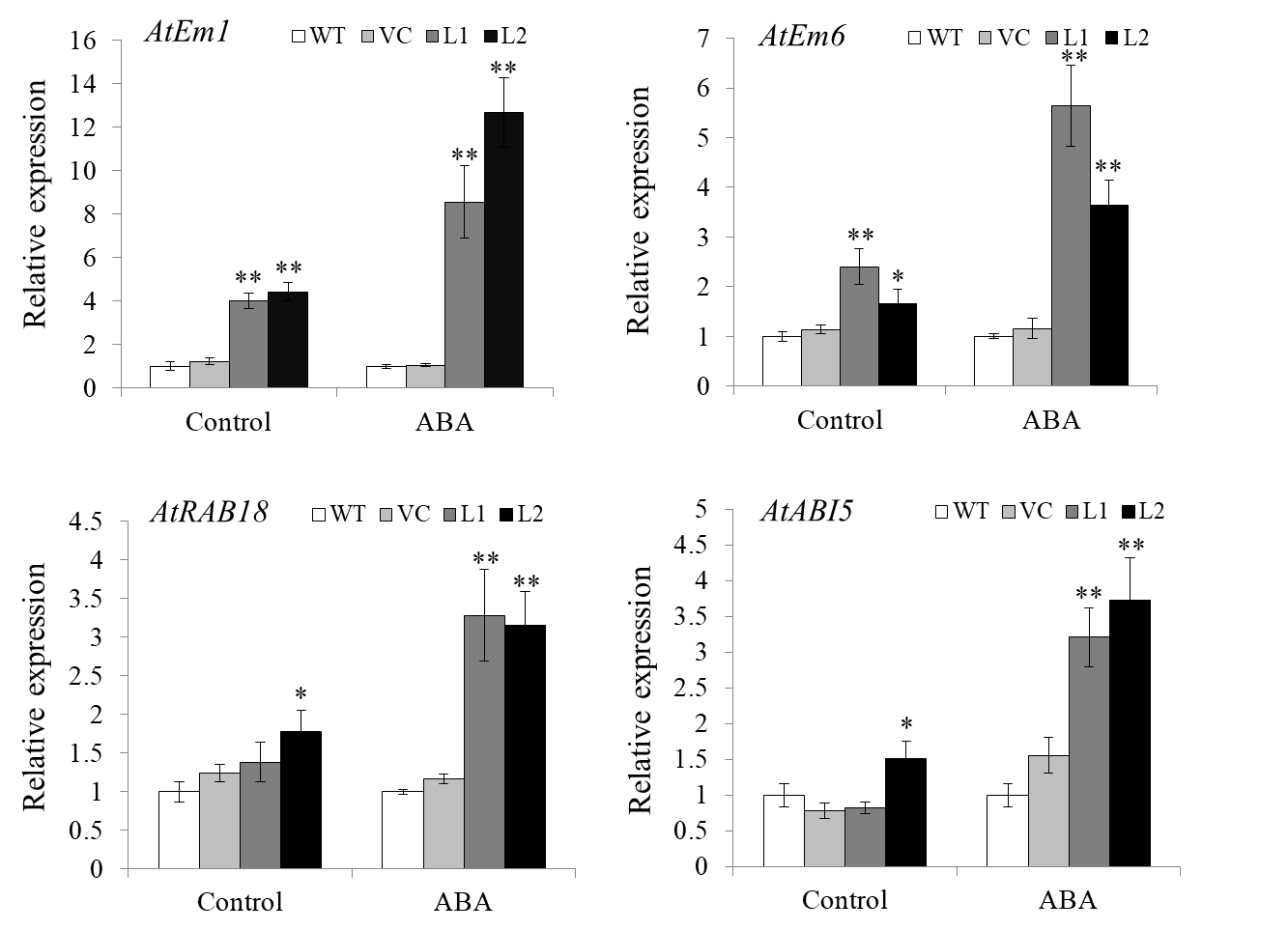


**Figure S1.** Expression of genes involved in ABA responses in *TaSAP7-A* transgenic *Arabidopsis*. Relative transcript levels of genes were determined by using RNA isolated from germinated seeds at 5 d after stratification on MS medium with or without 0.5 μM ABA. *AtEm1/6* = *Arabidopsis* late embryogenesis abundant 1/6. *AtRAB18* = Responsive to ABA 18. *AtABI5* = Abscisic acid-insensitive 5. The histogram represents mean ± SE of three biological replicates.

Figure S2.


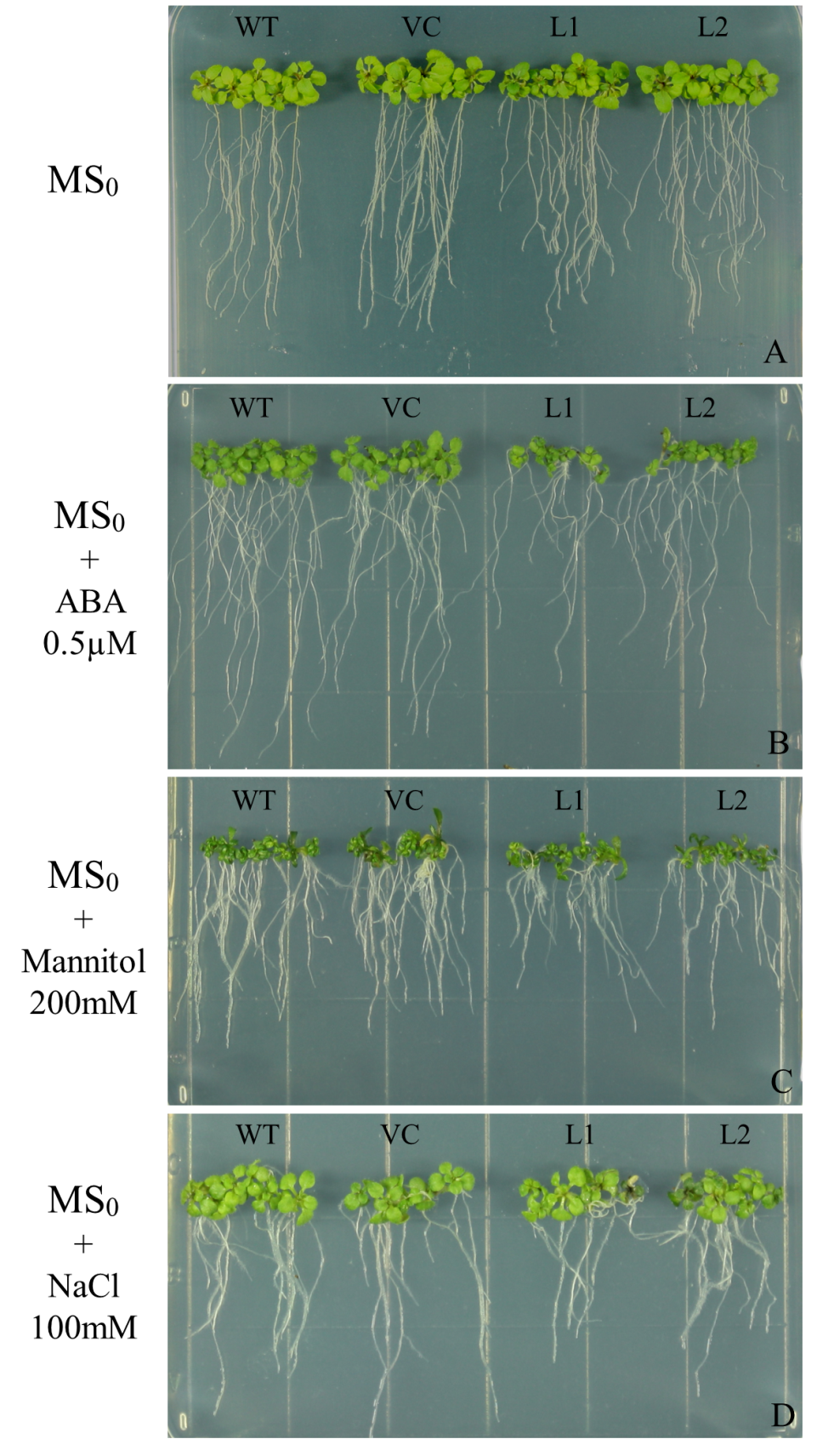


**Figure S2.** Effect of abiotic stress on root growth of *TaSAP7-A* transgenic *Arabidopsis*. The transgenic and control seeds were plated on MS medium supplemented with ABA (0.5 μM), mannitol (200 mM) and NaCl (100 mM), respectively. After stratification at 4°C for 2 d, the plates were incubated in vertical position to allow root growth for 15 d.

Figure S3.


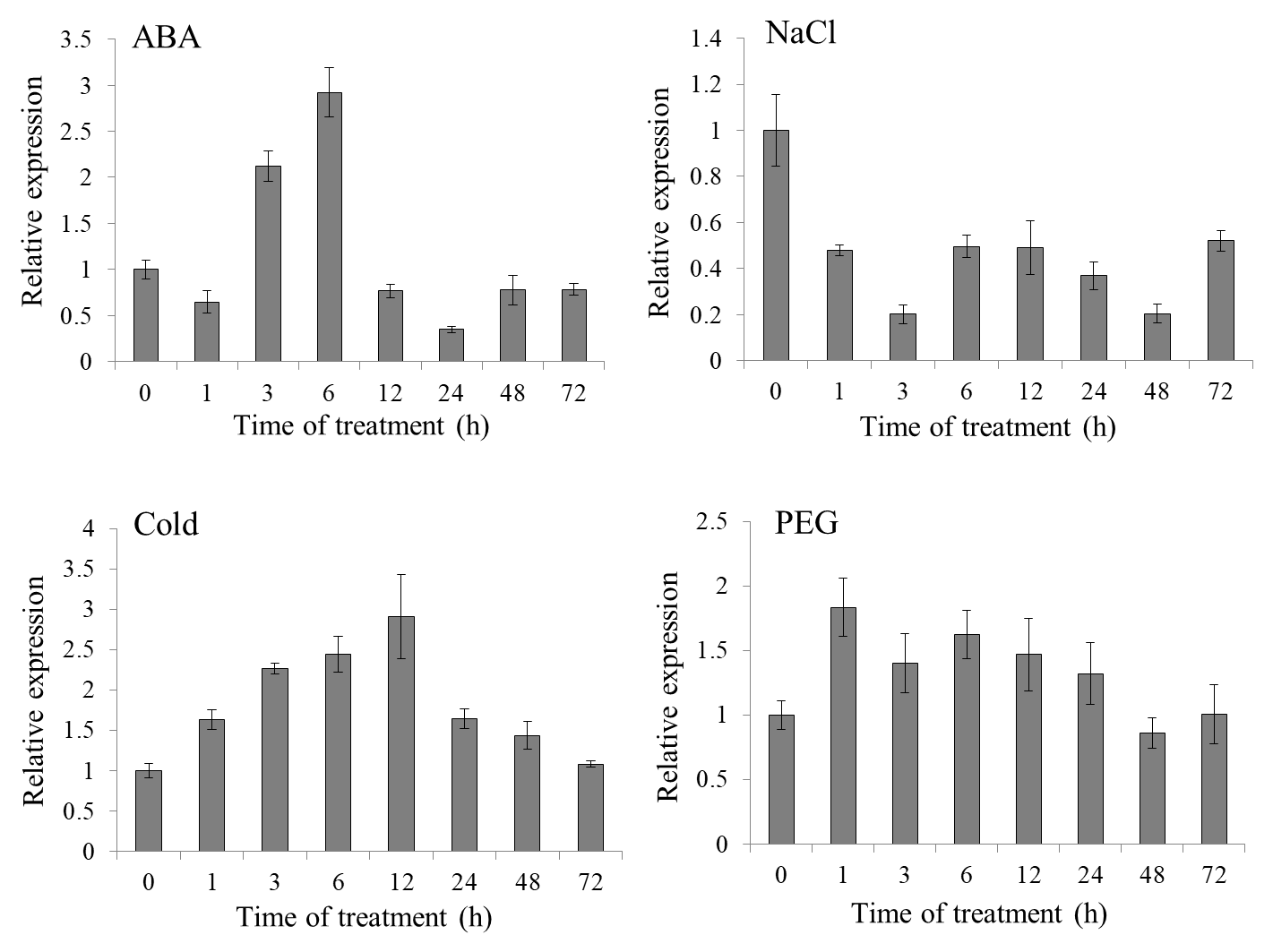


**Figure S3.** Expression patterns of *TaS10B* in wheat under abiotic stresses. Expression patterns of *TaS10B* in wheat seedlings exposed to 50 µM ABA, under salt stress (250 mM NaCl), osmotic stress (16.1% PEG-6000) and cold stress (4°C), respectively. The histogram represents mean ± SE of three biological replicates.

Figure S4.


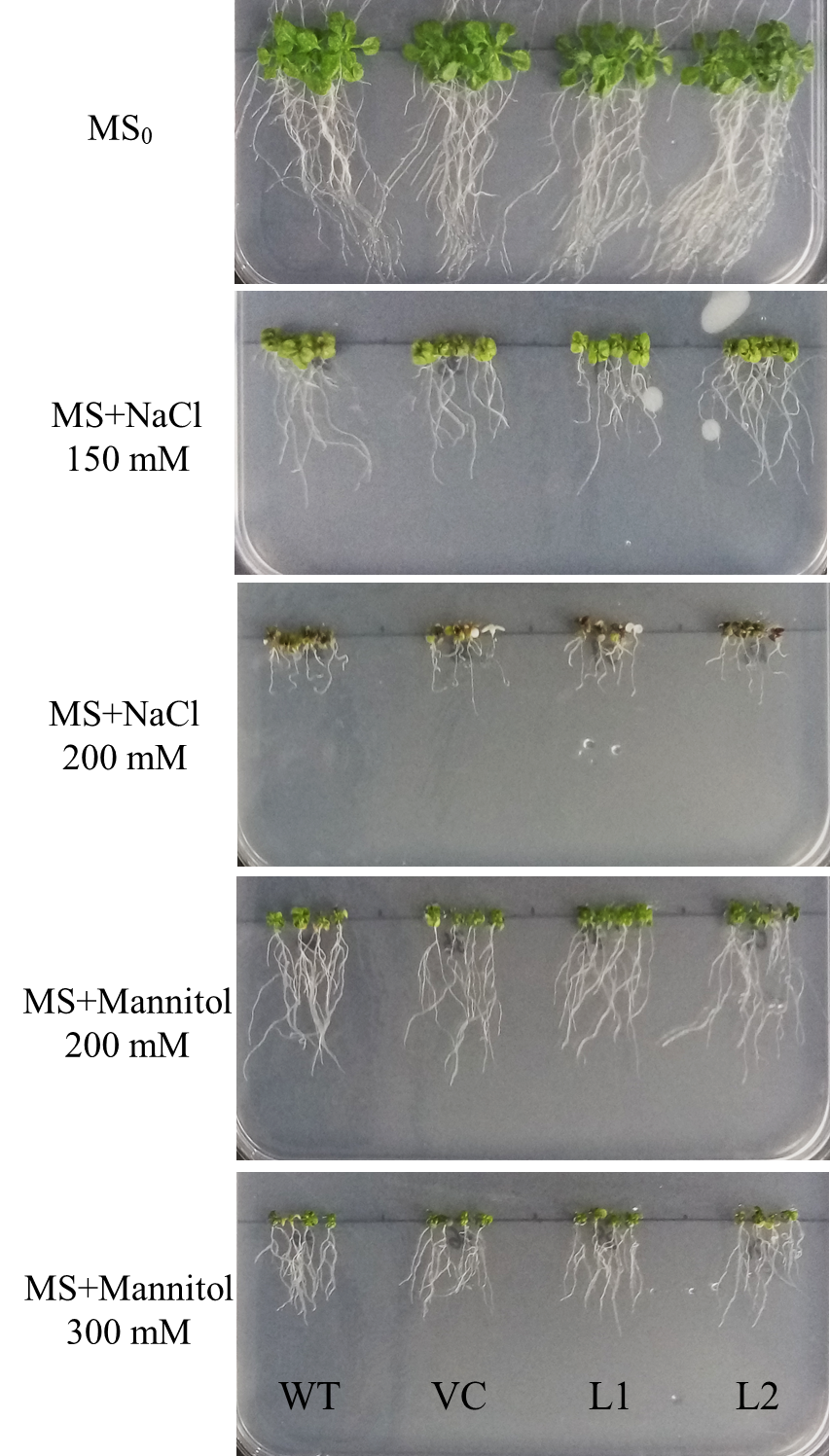


**Figure S4.** Effect of abiotic stress to *TaSAP7-A* transgenic *Arabidopsis* seedlings. The surface-sterilized transgenic and control seeds were placed on MS medium and grown vertically for 7d. The 7d seedlings were transplanted to MS medium with NaCl (150 mM and 200 mM) and mannitol (200 mM and 300 mM) for 7 d.
