# Supplementary material for: Cloning and Characterization of TaSAP7-A, a Member of the Stress-Associated Protein Family in Common Wheat
Source: Front Plant Sci. 2021 Mar 22;12:609351. doi: 10.3389/fpls.2021.609351 (PMC8020846; doi:10.3389/fpls.2021.609351)
Supplement: Supplementary file 2 [file Data_Sheet_2.doc]

**TABLE S1 The information of 32 highly diverse wheat accessions and their haplotypes of *TaSAP7-A***

| Number | Accession | Origin | Haplotype |
| --- | --- | --- | --- |
| 1 | PANDAS | Italy | *Hap-5A-1* |
| 2 | An85 Zhong124-1 | Beijing | *Hap-5A-1* |
| 3 | Yanzhan 1 | Henan | *Hap-5A-1* |
| 4 | Bawangbian | Hebei | *Hap-5A-1* |
| 5 | Beijing 10 | Beijing | *Hap-5A-1* |
| 6 | Beijing 14 | Beijing | *Hap-5A-3* |
| 7 | Cangzhouxiaomai | Hebei | *Hap-5A-3* |
| 8 | Changwu 131 | Shaanxi | *Hap-5A-2* |
| 9 | Chang 6878 | Shanxi | *Hap-5A-1* |
| 10 | Dali 1 | Shaanxi | *Hap-5A-1* |
| 11 | Dan R8093 | Beijing | *Hap-5A-2* |
| 12 | Fengkang 13 | Beijing | *Hap-5A-1* |
| 13 | Jimai 41 | Hebei | *Hap-5A-1* |
| 14 | Jimai 6 | Hebei | *Hap-5A-1* |
| 15 | Jin 2148-7 | Fujian | *Hap-5A-2* |
| 16 | Jinghe 8922 | Beijing | *Hap-5A-1* |
| 17 | Linkang 5108 | Shanxi | *Hap-5A-1* |
| 18 | Baiqimai | Gansu | *Hap-5A-2* |
| 19 | Changle 5 | Shandong | *Hap-5A-1* |
| 20 | Hongheshang | Shanxi | *Hap-5A-1* |
| 21 | Beijing 8686 | Beijing | *Hap-5A-1* |
| 22 | 04-044 | Beijing | *Hap-5A-1* |
| 23 | 04-030 | Beijing | *Hap-5A-1* |
| 24 | Chun 22 9th-25 | CIMMITY | *Hap-5A-1* |
| 25 | Ziganbaimangxian | Henan | *Hap-5A-1* |
| 26 | Jingpin 10 | Beijing | *Hap-5A-1* |
| 27 | Chun 04 9th-5-1 | CIMMITY | *Hap-5A-1* |
| 28 | Chun 45 9th-50-1 | CIMMITY | *Hap-5A-1* |
| 29 | Neixiang 188 | Henan | *Hap-5A-3* |
| 30 | Jing 411 | Beijing | *Hap-5A-1* |
| 31 | Chinese Spring | Sichuan | *Hap-5A-2* |
| 32 | Baicaomai | Henan | *Hap-5A-1* |

**TABLE S2** **The information of *TaSAP7-A* three haplotypes**

| Site | *Hap-5A-1* | *Hap-5A-2* | *Hap-5A-3* |
| --- | --- | --- | --- |
| -2721 | - | T | T |
| -2661 | G | A | A |
| -2608 | C | T | T |
| -2587 | T | C | C |
| -2356 | T | C | C |
| -2277 | G | - | - |
| -2268 | A | G | G |
| -2177 | G | C | C |
| -2044 | C | C | T |
| -1861 | G | A | A |
| -1722 | A | C | C |
| -1684 | A | - | - |
| -1668 | G | C | C |
| -1207 | C | A | A |
| -1185 | C | A | A |
| -1125 | G | A | A |
| -1063 | T | C | C |
| -1057 | A | G | G |
| -1045 | G | A | A |
| -1014 | T | G | G |
| -905 | G | T | T |
| -879 | - | A | A |
| -871 | C | A | A |
| -809 | A | T | T |
| -783 | C | A | A |
| -776 | A | G | G |
| -719 | C | T | T |
| -685 | G | A | A |
| -667 | G | A | A |
| -633 | T | A | A |
| -505 | G | A | A |
| -464 | A | G | G |
| -419 | C | G | G |
| -367 | G | C | C |
| -354 | T | C | C |
| -58 | A | C | C |
| -31 | C | G | G |
| 235 | A | G | G |
| 512 | T | C | C |

***TABLE S3 The information of 323 wheat accessions and their genotypes of TaSAP7-A***

| Number | Accession | Type | Origin | Haplotype | Number | Accession | Type | Origin | Haplotype |
| --- | --- | --- | --- | --- | --- | --- | --- | --- | --- |
| 1 | Drysdale | M | [Australia](http://www.youdao.com/w/Australia/#keyfrom=E2Ctranslation) | *Hap-5A-1* | 163 | Jimai 2 | M | Hebei | *Hap-5A-2* |
| 2 | SALGEMMA | M | [Italy](http://www.youdao.com/w/Italy/#keyfrom=E2Ctranslation) | *Hap-5A-3* | 164 | jimai 30 | M | Hebei | *Hap-5A-1* |
| 3 | Bainong 160 | M | Henan | *Hap-5A-2* | 165 | Jimai 32 | M | Hebei | *Hap-5A-2* |
| 4 | Bo'ai 7023 | M | Henan | *Hap-5A-2* | 166 | Jimai 38 | M | Hebei | *Hap-5A-1* |
| 5 | Dali 1 | M | Shaanxi | *Hap-5A-1* | 167 | Jimai 41 | M | Hebei | *Hap-5A-1* |
| 6 | Dali 52 | M | Shaanxi | *Hap-5A-1* | 168 | Jimai 6 | M | Hebei | *Hap-5A-1* |
| 7 | Fanmai 8 | M | Henan | *Hap-5A-1* | 169 | Jimai 9 | M | Hebei | *Hap-5A-3* |
| 8 | Fengchan 1 | M | Shaanxi | *Hap-5A-3* | 170 | Jimai 1 | M | Hebei | *Hap-5A-3* |
| 9 | Fengchan 3 | M | Shaanxi | *Hap-5A-1* | 171 | Jishen 5099 | M | Hebei | *Hap-5A-1* |
| 10 | Fengyou 5 | M | Beijing | *Hap-5A-1* | 172 | Jian 26 | M | Beijing | *Hap-5A-3* |
| 11 | Fuzhuang 30 | M | Shaanxi | *Hap-5A-1* | 173 | Jinguang | M | Shaanxi | *Hap-5A-1* |
| 12 | Han05-5092 | AL | Hebei | *Hap-5A-1* | 174 | Jin 2148-7 | M | Shanxi | *Hap-5A-2* |
| 13 | Han 6172 | M | Hebei | *Hap-5A-2* | 175 | Jinmai 13 | M | Shanxi | *Hap-5A-1* |
| 14 | Handan 6050 | M | Hebei | *Hap-5A-3* | 176 | Jinmai 17 | M | Shanxi | *Hap-5A-1* |
| 15 | Heng 216 | M | Hebei | *Hap-5A-2* | 177 | Jinmai 33 | M | Shanxi | *Hap-5A-1* |
| 16 | Heng 4399 | M | Hebei | *Hap-5A-2* | 178 | Jinmai 39 | M | Shanxi | *Hap-5A-1* |
| 17 | Heng 5229 | M | Hebei | *Hap-5A-1* | 179 | Jinmai 44 | M | Shanxi | *Hap-5A-1* |
| 18 | Heng 7228 | M | Hebei | *Hap-5A-1* | 180 | Jinmai 47 | M | Shanxi | *Hap-5A-1* |
| 19 | Heng 95 guan 26 | M | Hebei | *Hap-5A-1* | 181 | Jinmai 50 | M | Shanxi | *Hap-5A-1* |
| 20 | Hengguan 35 | M | Hebei | *Hap-5A-2* | 182 | Jinmai 51 | M | Shanxi | *Hap-5A-1* |
| 21 | Hengmai 2 | AL | Hebei | *Hap-5A-1* | 183 | Jinmai 53 | M | Shanxi | *Hap-5A-1* |
| 22 | Hengshui 6404 | M | Hebei | *Hap-5A-1* | 184 | Jinmai 54 | M | Shanxi | *Hap-5A-1* |
| 23 | Hengyou 18 | M | Hebei | *Hap-5A-1* | 185 | Jinmai 57 | M | Shanxi | *Hap-5A-1* |
| 24 | Huaimai 18 | M | Jangsu | *Hap-5A-1* | 186 | Jinmai 63 | M | Shanxi | *Hap-5A-1* |
| 25 | Huaimai 25 | M | Jangsu | *Hap-5A-1* | 187 | Jinmai 68 | M | Shanxi | *Hap-5A-1* |
| 26 | Huaishu 10 | M | Jangsu | *Hap-5A-1* | 188 | Jinmai 72 | M | Shanxi | *Hap-5A-3* |
| 27 | Lantian 15 | M | Gansu | *Hap-5A-1* | 189 | Jinmai 79 | M | Shanxi | *Hap-5A-3* |
| 28 | Liangxing 99 | M | Shandong | *Hap-5A-2* | 190 | Jinmai 91 | M | Shanxi | *Hap-5A-1* |
| 29 | Lovrin 10 | M | Rumania | *Hap-5A-1* | 191 | Jinnong 207 | M | Shanxi | *Hap-5A-2* |
| 30 | Luohan 11 | M | Henan | *Hap-5A-1* | 192 | Jintai 102 | M | Shanxi | *Hap-5A-2* |
| 31 | Luohan 13 | M | Henan | *Hap-5A-1* | 193 | Jintai 114 | M | Shanxi | *Hap-5A-1* |
| 32 | Luohan 2 | M | Henan | *Hap-5A-1* | 194 | Jintai 1310 | M | Shanxi | *Hap-5A-1* |
| 33 | Luohan 3 | M | Henan | *Hap-5A-1* | 195 | Jintai 182 | M | Shanxi | *Hap-5A-1* |
| 34 | Luohan 6 | M | Henan | *Hap-5A-1* | 196 | Jing 411 | M | Beijing | *Hap-5A-1* |
| 35 | Luohan 7 | M | Henan | *Hap-5A-1* | 197 | Jingdong 82 Dong 307 | M | Beijing | *Hap-5A-1* |
| 36 | Luohan 8 | M | Henan | *Hap-5A-1* | 198 | Jingdong 83 Dong 65 | M | Beijing | *Hap-5A-1* |
| 37 | Luohan 9 | M | Henan | *Hap-5A-1* | 199 | Jingdong 8 | M | Beijing | *Hap-5A-1* |
| 38 | Luomai 21 | M | Henan | *Hap-5A-1* | 200 | Jinghe 8922 | M | Beijing | *Hap-5A-1* |
| 39 | Luomai 23 | M | Henan | *Hap-5A-1* | 201 | Jinghua 1 | M | Beijing | *Hap-5A-1* |
| 40 | Luonong 10 | M | Shaanxi | *Hap-5A-1* | 202 | Jingnong 79-15 | M | Beijing | *Hap-5A-1* |
| 41 | Luoyang 8628 | M | Henan | *Hap-5A-1* | 203 | Jingnong 80 Jian 107 | M | Beijing | *Hap-5A-1* |
| 42 | Luomai 8 | M | Henan | *Hap-5A-2* | 204 | Jingnong 84-6786 | M | Beijing | *Hap-5A-2* |
| 43 | Luomai 9 | M | Henan | *Hap-5A-1* | 205 | Jingpin 11 | M | Beijing | *Hap-5A-1* |
| 44 | Luoyou 7 | M | Henan | *Hap-5A-1* | 206 | Jingpin 30 | M | Beijing | *Hap-5A-1* |
| 45 | Qingchun 1 | M | Shaanxi | *Hap-5A-3* | 207 | Jingpin 3 | M | Beijing | *Hap-5A-1* |
| 46 | Qingchun 2 | M | Shaanxi | *Hap-5A-3* | 208 | Jingshuang 16 | M | Beijing | *Hap-5A-1* |
| 47 | Qingshan 843 | M | Gansu | *Hap-5A-1* | 209 | Jingshuang 2 | M | Beijing | *Hap-5A-3* |
| 48 | Shi 4185 | M | Hebei | *Hap-5A-1* | 210 | Jingxuan 20 | M | Beijing | *Hap-5A-1* |
| 49 | Shijiazhuang 407 | M | Hebei | *Hap-5A-1* | 211 | Jingxuan 25 | M | Beijing | *Hap-5A-1* |
| 50 | Shijiazhuang 8 | M | Hebei | *Hap-5A-1* | 212 | Jingyan 85 Jian 28 | M | Beijing | *Hap-5A-1* |
| 51 | Shimai 12 | M | Hebei | *Hap-5A-1* | 213 | Kenong 199 | M | Hebei | *Hap-5A-1* |
| 52 | Shimai 13 | M | Hebei | *Hap-5A-1* | 214 | Keyi 26 | M | Beijing | *Hap-5A-1* |
| 53 | Shimai 15 | M | Hebei | *Hap-5A-2* | 215 | Keyi 29 | M | Beijing | *Hap-5A-1* |
| 54 | Shimai 18 | M | Hebei | *Hap-5A-1* | 216 | Lin 138 | M | Shanxi | *Hap-5A-2* |
| 55 | Shimai 19 | M | Hebei | *Hap-5A-1* | 217 | Linfen 8050 | M | Shanxi | *Hap-5A-1* |
| 56 | Xuzhou 21 | M | Jangsu | *Hap-5A-1* | 218 | Linfeng 3 | M | Shanxi | *Hap-5A-1* |
| 57 | Xuzhou 6 | M | Jangsu | *Hap-5A-1* | 219 | Linfeng 518 | M | Shanxi | *Hap-5A-1* |
| 58 | Yanzhan 1 | M | Henan | *Hap-5A-1* | 220 | Linhan 5089 | M | Shanxi | *Hap-5A-1* |
| 59 | Yubao 1 | M | Henan | *Hap-5A-1* | 221 | Linhan 5367 | M | Shanxi | *Hap-5A-2* |
| 60 | Yumai 13 | M | Henan | *Hap-5A-2* | 222 | Linhan 6105 | M | Shanxi | *Hap-5A-1* |
| 61 | Yumai 18 | M | Henan | *Hap-5A-1* | 223 | Linhan 6 | M | Shanxi | *Hap-5A-1* |
| 62 | Yumai 29 | M | Henan | *Hap-5A-1* | 224 | Linhan 917 | M | Shanxi | *Hap-5A-1* |
| 63 | Yumai 2 | M | Henan | *Hap-5A-3* | 225 | Linhan 935 | M | Shanxi | *Hap-5A-1* |
| 64 | Yumai 38 | M | Henan | *Hap-5A-1* | 226 | Linkang 5108 | AL | Shanxi | *Hap-5A-1* |
| 65 | Yumai 47 | M | Henan | *Hap-5A-3* | 227 | Longjian 196 | M | Gansu | *Hap-5A-1* |
| 66 | Yumai 48 | M | Henan | *Hap-5A-3* | 228 | Longjian 294 | M | Gansu | *Hap-5A-1* |
| 67 | Yumai 8 | M | Henan | *Hap-5A-3* | 229 | Lude 1 | M | Shandong | *Hap-5A-1* |
| 68 | Yunong 416 | M | Henan | *Hap-5A-2* | 230 | Lumai 14 | M | Shandong | *Hap-5A-1* |
| 69 | Yunong 949 | M | Henan | *Hap-5A-1* | 231 | Lumai 15 | M | Shandong | *Hap-5A-3* |
| 70 | Yuzhan 4 | M | Henan | *Hap-5A-2* | 232 | Lumai 17 | M | Shandong | *Hap-5A-1* |
| 71 | Zhoumai 16 | M | Henan | *Hap-5A-1* | 233 | Lumai 19 | M | Shandong | *Hap-5A-2* |
| 72 | Zhoumai 18 | M | Henan | *Hap-5A-1* | 234 | Lumai 23 | M | Shandong | *Hap-5A-1* |
| 73 | Zhoumai 22 | M | Henan | *Hap-5A-1* | 235 | Lumai 3 | M | Shandong | *Hap-5A-1* |
| 74 | Zhoumai 23 | M | Henan | *Hap-5A-1* | 236 | Lumai 5 | M | Shandong | *Hap-5A-3* |
| 75 | Shite 14 | M | Hebei | *Hap-5A-1* | 237 | Lumai 8 | M | Shandong | *Hap-5A-3* |
| 76 | Shiyou 17 | M | Hebei | *Hap-5A-1* | 238 | Lunkang 7 | M | Beijing | *Hap-5A-1* |
| 77 | Shiyou 20 | M | Hebei | *Hap-5A-1* | 239 | Lunxuan 987 | M | Beijing | *Hap-5A-2* |
| 78 | Wanmai 19 | M | Anhui | *Hap-5A-1* | 240 | Mazhamai | L | Shaanxi | *Hap-5A-1* |
| 79 | Wenmai 6 | M | Henan | *Hap-5A-1* | 241 | Mingxian 169 | M | Shanxi | *Hap-5A-1* |
| 80 | Xi'an 8 | M | Shaanxi | *Hap-5A-3* | 242 | Ningdong 11 | M | Ningxia | *Hap-5A-1* |
| 81 | Xinong 1018 | M | Shaanxi | *Hap-5A-1* | 243 | Nongda 135 | M | Beijing | *Hap-5A-1* |
| 82 | Xinong 189 | M | Shaanxi | *Hap-5A-2* | 244 | Nongda 146 | M | Beijing | *Hap-5A-1* |
| 83 | Xinong 219 | M | Shaanxi | *Hap-5A-2* | 245 | Nongda 155 | M | Beijing | *Hap-5A-1* |
| 84 | Xinong 318 | M | Shaanxi | *Hap-5A-2* | 246 | Nongda 183 | M | Beijing | *Hap-5A-1* |
| 85 | Xinong 6028 | M | Shaanxi | *Hap-5A-3* | 247 | Nongda 20074 | M | Beijing | *Hap-5A-1* |
| 86 | Xinong 688 | M | Shaanxi | *Hap-5A-1* | 248 | Nongda 311 | M | Beijing | *Hap-5A-1* |
| 87 | Xinong 928 | M | Shaanxi | *Hap-5A-1* | 249 | Nongda 3195 | M | Beijing | *Hap-5A-1* |
| 88 | Xinong 9106 | M | Shaanxi | *Hap-5A-1* | 250 | Nongda 33 | M | Beijing | *Hap-5A-1* |
| 89 | Xinmai 296 | M | Shandong | *Hap-5A-1* | 251 | Nongda 36 | M | Beijing | *Hap-5A-1* |
| 90 | Jimai 19 | M | Shandong | *Hap-5A-1* | 252 | Nongda 81146 | AL | Beijing | *Hap-5A-1* |
| 91 | Jimai 20 | M | Shandong | *Hap-5A-1* | 253 | Pingliang 35 | M | Gansu | *Hap-5A-1* |
| 92 | Jimai 21 | M | Shandong | *Hap-5A-1* | 254 | Pingyang 348 | AL | Shanxi | *Hap-5A-1* |
| 93 | Jimai 22 | M | Shandong | *Hap-5A-1* | 255 | Qinmai 3 | M | Shaanxi | *Hap-5A-1* |
| 94 | Jimai 4 | M | Shandong | *Hap-5A-1* | 256 | Qinmai 7 | M | Shaanxi | *Hap-5A-1* |
| 95 | Jinan 10 | M | Shandong | *Hap-5A-1* | 257 | Qingfeng 1 | M | Gansu | *Hap-5A-1* |
| 96 | Jinan 13 | M | Shandong | *Hap-5A-3* | 258 | Shannongfu 63 | M | Shandong | *Hap-5A-1* |
| 97 | Jinan 2 | M | Shandong | *Hap-5A-1* | 259 | Shannongyoumai 2 | M | Shandong | *Hap-5A-1* |
| 98 | Jining 3 | M | Shandong | *Hap-5A-1* | 260 | Shanyou 2 | M | Henan | *Hap-5A-1* |
| 99 | Han 4589 | M | Hebei | *Hap-5A-3* | 261 | Shan225-9 | M | Shaanxi | *Hap-5A-1* |
| 100 | Heng 136 | M | Hebei | *Hap-5A-1* | 262 | Shaan 229 | M | Shaanxi | *Hap-5A-1* |
| 101 | Jimai 6 | M | Henan | *Hap-5A-1* | 263 | Shaanhan 8675 | M | Shaanxi | *Hap-5A-1* |
| 102 | Luomai 22 | M | Shandong | *Hap-5A-1* | 264 | Shaanhe 6 | M | Shaanxi | *Hap-5A-1* |
| 103 | Qingmai 7 | M | Shandong | *Hap-5A-3* | 265 | Shaannong 1 | M | Shaanxi | *Hap-5A-1* |
| 104 | Xinong 1043 | M | Shaanxi | *Hap-5A-1* | 266 | Shaannong 2 | M | Shaanxi | *Hap-5A-3* |
| 105 | Hongliang 4 | M | Beijing | *Hap-5A-3* | 267 | Triumph | M | America | *Hap-5A-2* |
| 106 | Jinmai 16 | M | Shanxi | *Hap-5A-1* | 268 | Shuangfengshou | M | Shaanxi | *Hap-5A-3* |
| 107 | Jinmai 25 | M | Shanxi | *Hap-5A-1* | 269 | Shunmai 1718 | M | Shanxi | *Hap-5A-2* |
| 108 | Yunhan 22-33 | M | Shanxi | *Hap-5A-1* | 270 | Silenghonghulutou | L | Hebei | *Hap-5A-1* |
| 109 | An 86 Zhong 17 | AL | Beijing | *Hap-5A-1* | 271 | Tai 13606 | M | Shanxi | *Hap-5A-1* |
| 110 | Bawangbian | L | Hebei | *Hap-5A-1* | 272 | Tai 712 | M | Shanxi | *Hap-5A-1* |
| 111 | Baicaomai | L | Henan | *Hap-5A-1* | 273 | Taiyuan 566 | AL | Shanxi | *Hap-5A-1* |
| 112 | Baiqimai | L | Gansu | *Hap-5A-2* | 274 | Taiyuan 633 | M | Shanxi | *Hap-5A-1* |
| 113 | Baitutou | L | Shandong | *Hap-5A-1* | 275 | Taishan 23 | M | Shandong | *Hap-5A-1* |
| 114 | Baolin 9 | M | Shaanxi | *Hap-5A-1* | 276 | Taishan 24 | M | Shandong | *Hap-5A-1* |
| 115 | Baomai 5 | M | Shaanxi | *Hap-5A-1* | 277 | Weimai 4 | M | Shaanxi | *Hap-5A-1* |
| 116 | Beijing 837 | M | Beijing | *Hap-5A-3* | 278 | Xifeng 16 | M | Gansu | *Hap-5A-1* |
| 117 | Beijing 8686 | M | Beijing | *Hap-5A-1* | 279 | Xifeng 20 | M | Gansu | *Hap-5A-3* |
| 118 | Beijing 8694 | M | Beijing | *Hap-5A-3* | 280 | Xifeng 9 | M | Gansu | *Hap-5A-1* |
| 119 | Beinong 2 | M | Beijing | *Hap-5A-2* | 281 | Xiaobaimai | L | Shanxi | *Hap-5A-1* |
| 120 | Bima 1 | M | Shaanxi | *Hap-5A-1* | 282 | Xiaoshan 8 | AL | Beijing | *Hap-5A-1* |
| 121 | Cangmai 6001 | M | Hebei | *Hap-5A-2* | 283 | Xindong 20 | M | Xinjiang | *Hap-5A-1* |
| 122 | Cangmai 6005 | M | Hebei | *Hap-5A-1* | 284 | Xindong 22 | M | Xinjiang | *Hap-5A-1* |
| 123 | Cangzhouxiaomai | L | Hebei | *Hap-5A-3* | 285 | Yannong 19 | M | Shandong | *Hap-5A-2* |
| 124 | Changle 5 | M | Shandong | *Hap-5A-1* | 286 | Yannong 21 | M | Shandong | *Hap-5A-2* |
| 125 | Chang 4640 | M | Shanxi | *Hap-5A-1* | 287 | Yanan 15 | M | Shaanxi | *Hap-5A-1* |
| 126 | Chang 4738 | M | Shanxi | *Hap-5A-2* | 288 | Yanda 1817 | M | Beijing | *Hap-5A-1* |
| 127 | Chang 4853 | M | Shanxi | *Hap-5A-1* | 289 | Yuandong 3 | M | Beijing | *Hap-5A-3* |
| 128 | Chang 5259 | M | Shanxi | *Hap-5A-2* | 290 | Yuandong 834 | AL | Beijing | *Hap-5A-1* |
| 129 | Chang 6154 | M | Shanxi | *Hap-5A-1* | 291 | Yuandong 847 | AL | Beijing | *Hap-5A-2* |
| 130 | Chang 6359 | M | Shanxi | *Hap-5A-2* | 292 | Yuandong 856 | AL | Beijing | *Hap-5A-3* |
| 131 | Chang 6452 | M | Shanxi | *Hap-5A-1* | 293 | Yunhan 102 | M | Shanxi | *Hap-5A-1* |
| 132 | Chang 6794 | M | Shanxi | *Hap-5A-1* | 294 | Yunhan 115 | M | Shanxi | *Hap-5A-1* |
| 133 | Chang 6878 | M | Shanxi | *Hap-5A-1* | 295 | Yunhan 2028 | AL | Shanxi | *Hap-5A-1* |
| 134 | Chang 8744 | M | Shanxi | *Hap-5A-2* | 296 | Yunhan 20410 | M | Shanxi | *Hap-5A-1* |
| 135 | Changmai 6135 | M | Shanxi | *Hap-5A-1* | 297 | Yunhan 21-30 | M | Shanxi | *Hap-5A-1* |
| 136 | Changwu 131 | M | Shaanxi | *Hap-5A-2* | 298 | Yunhan 23-35 | M | Shanxi | *Hap-5A-1* |
| 137 | Changwu 134 | M | Shaanxi | *Hap-5A-2* | 299 | Yunhan 618 | M | Shanxi | *Hap-5A-2* |
| 138 | Changwu 89(1)3-4 | M | Shaanxi | *Hap-5A-2* | 300 | Yunhan 719 | M | Shanxi | *Hap-5A-1* |
| 139 | Changzhi 516 | M | Shanxi | *Hap-5A-1* | 301 | Yunhan 805 | M | Shanxi | *Hap-5A-1* |
| 140 | Changzhi 620 | M | Shanxi | *Hap-5A-1* | 302 | Zaosui 21 | AL | Beijing | *Hap-5A-1* |
| 141 | Dan R8043 | AL | Beijing | *Hap-5A-2* | 303 | Zaosui 65 | AL | Beijing | *Hap-5A-1* |
| 142 | Dan R8093 | AL | Beijing | *Hap-5A-2* | 304 | Zaosui 66 | AL | Beijing | *Hap-5A-1* |
| 143 | Dan R8108 | AL | Beijing | *Hap-5A-2* | 305 | Early premium | M | America | *Hap-5A-1* |
| 144 | Dan R8194 | AL | Beijing | *Hap-5A-2* | 306 | Zhangdong 29 | M | Gansu | *Hap-5A-1* |
| 145 | Dongxie 2 | M | Beijing | *Hap-5A-2* | 307 | Zhengfeng 9962 | M | Henan | *Hap-5A-1* |
| 146 | Fengkang 13 | M | Beijing | *Hap-5A-1* | 308 | Zhengzhou 24 | M | Henan | *Hap-5A-1* |
| 147 | Hanxuan 10 | M | Shanxi | *Hap-5A-1* | 309 | Zhong 7902 | AL | Beijing | *Hap-5A-1* |
| 148 | Hanxuan 11 | M | Shanxi | *Hap-5A-1* | 310 | Zhong 86 I-50455 | AL | Beijing | *Hap-5A-1* |
| 149 | Hanxuan 12 | M | Shanxi | *Hap-5A-1* | 311 | Zhongda 86-Jian 2 | AL | Beijing | *Hap-5A-1* |
| 150 | Hanxuan 1 | M | Shanxi | *Hap-5A-1* | 312 | Zhongda 91-Pin 9 | M | Beijing | *Hap-5A-1* |
| 151 | Hanxuan 2 | M | Shanxi | *Hap-5A-1* | 313 | Zhongda 92-Jian 49 | M | Beijing | *Hap-5A-3* |
| 152 | Hanxuan 3 | M | Shanxi | *Hap-5A-1* | 314 | Zhongda 92-Pin 8 | M | Beijing | *Hap-5A-1* |
| 153 | Heimangmai | L | Hebei | *Hap-5A-1* | 315 | Zhonghan 110 | M | Beijing | *Hap-5A-3* |
| 154 | Hongheshang | L | Shanxi | *Hap-5A-1* | 316 | Zhongmai 175 | M | Beijing | *Hap-5A-1* |
| 155 | Hulutou | L | Hebei | *Hap-5A-1* | 317 | Zhongmai 9 | M | Beijing | *Hap-5A-1* |
| 156 | Huapei 6 | M | Henan | *Hap-5A-1* | 318 | Zhongsu 68 | M | Beijing | *Hap-5A-2* |
| 157 | Huabei 187 | M | Beijing | *Hap-5A-1* | 319 | Zhongyin 6 | M | Beijing | *Hap-5A-2* |
| 158 | Ji 92-5203 | AL | Hebei | *Hap-5A-2* | 320 | Zhongyou 9507 | M | Beijing | *Hap-5A-1* |
| 159 | Jimai 10 | M | Hebei | *Hap-5A-1* | 321 | Zhongzuo 60064 | AL | Beijing | *Hap-5A-1* |
| 160 | Jimai 22 | M | Hebei | *Hap-5A-1* | 322 | Zhongzuo 60115 | AL | Beijing | *Hap-5A-1* |
| 161 | Jimai 26 | M | Hebei | *Hap-5A-1* | 323 | Ziganbaimangxian | L | Henan | *Hap-5A-1* |
| 162 | Jimai 29 | M | Hebei | *Hap-5A-1* |  |  |  |  |  |

L, Landraces; AL, Advanced lines; M, Modern varieties.
